# Thyroid-bed schwannoma mimicking a thyroid neoplasm: a case report

**DOI:** 10.3389/fmed.2026.1982014

**Published:** 2026-10-15

**Authors:** Mingyu Wang, Boyang Yu

**Affiliations:** Department of Medical Ultrasound, West China Hospital, Sichuan University, Chengdu, China

**Keywords:** core needle biopsy, fine-needle aspiration, neck schwannoma, thyroid-bed lesion, thyroidectomy

## Abstract

Intrathyroidal schwannomas are rare, and thyroid-bed schwannomas are scarcer, with merely a few case reports and tough preoperative differentiation. We report a 33-year-old female with a swallowing-movable left neck mass and normal thyroid function. Gray-scale B-mode ultrasound, color Doppler flow imaging and contrast-enhanced ultrasound showed showed a heterogeneously enhanced TI-RADS 3 lesion preoperatively suspected as a follicular neoplasm. Fine-needle aspiration (FNA) biopsy was performed, which failed to yield a definitive pathological diagnosis and yielded Bethesda category I. She received left lobectomy plus isthmectomy, and pathology confirmed thyroid-bed schwannoma sparing thyroid parenchyma; she recovered well with normal thyroid function postoperatively. Due to its low incidence, this tumor is often mistaken for an intrathyroidal nodule on ultrasound. Total surgical excision is the first-line therapy with good overall prognosis. Unlike intrathyroidal schwannomas, most thyroid-bed lesions can be enucleated alone without routine thyroidectomy; hemithyroidectomy is needed for dense adhesion, and total thyroidectomy is rarely required. Considering the low diagnostic value of FNA, multidisciplinary consultation of pathology, radiology and surgery is advised for lesions with atypical imaging, inconclusive FNA or ill-defined thyroid margins. Ultrasound-guided core needle biopsy (CNB) should be performed if needed to tailor proper treatment.

## Introduction

Schwannomas are benign neurogenic tumors arising from Schwann cells of nerve sheaths throughout the body. With an annual incidence of approximately 0.6 per 100,000 population, they most commonly occur on the flexor surfaces of the extremities, and roughly 25%-45% of cases are located in the neck region (1, 2). Primary schwannomas arising within the thyroid parenchyma are exceedingly rare, while thyroid-bed schwannomas are even rarer, with only 16 cases reported in the published literature to date (3, 4). Owing to non-specific features on both ultrasonography and computed tomography (CT), coupled with the limited diagnostic yield of fine-needle aspiration (FNA), most previously reported cases remain undiagnosed before surgery (2, 3, 5–14). Herein, we report a case of thyroid-bed schwannoma, which was preoperatively highly suspected to be a primary thyroid follicular neoplasm based on imaging findings. The final diagnosis was confirmed by postoperative histopathology following thyroid lobectomy.

## Case presentation

A 33-year-old female presented with a left thyroid mass incidentally detected during physical examination, with follow-up imaging demonstrating progressive lesion growth. Initial thyroid ultrasonography performed 9 years prior identified a nodule in the left thyroid lobe with no detailed documentation available. The patient was asymptomatic at that time and received no intervention, and she reported gradual enlargement of the neck mass over the intervening years. Follow-up thyroid ultrasonography performed 3 years before admission identified a large cystic and solid mixed echogenic mass occupying nearly the entire left thyroid lobe, with obscured visualization of normal gland parenchyma. The mass measured approximately 58 × 44 × 59 mm (estimated volume approximately 78.8 cm^3^), with relatively well-defined borders, regular morphology, heterogeneous internal echogenicity, and punctate linear blood flow signals within the lesion. Based on these imaging features, a follicular neoplasm was highly suspected. Although surgical intervention was indicated, the patient opted for close surveillance and deferred surgery for personal reasons. Repeat thyroid ultrasonography performed at an external hospital 12 days prior to admission revealed a cystic-solid nodule in the middle and lower portion of the left thyroid lobe. The nodule demonstrated regular morphology and relatively clear borders, measured approximately 61 × 44 × 50 mm (estimated volume approximately 70.3 cm^3^), and was predominantly solid in composition. Multiple punctate patchy hyperechoic foci with posterior acoustic shadowing were noted within the solid component, and the anteroposterior-to-transverse diameter ratio was less than 1. These findings were consistent with slow progression of the same lesion. The patient subsequently presented to our institution for further diagnosis and management. The patient denied a family history of neurofibromatosis, as well as histories of smoking, substance abuse, and excessive alcohol consumption. Physical examination detected a well-circumscribed, regularly shaped mass measuring approximately 6 × 4 × 5 cm in the left neck, which moved synchronously with deglutition. No enlarged cervical lymph nodes were palpable.

Findings from examinations performed at admission are summarized below. Thyroid function tests were normal. Gray-scale B-mode ultrasound (Figure 1A, B) demonstrated that the left thyroid lobe was occupied by a large mass located at the inferior portion adjacent to the peripheral capsule, measuring 62 × 35 × 47 mm (estimated volume approximately 53.4 cm^3^). The mass had an anteroposterior-to-transverse diameter ratio below 1, with slightly irregular shape and relatively clear border but no peripheral hypoechoic halo. It presented heterogeneous mixed cystic and solid internal echoes with mixed posterior acoustic features and coarse calcifications, capsular invasion could not be definitively excluded. Color Doppler imaging revealed scant intralesional blood flow signals within the mass, which was classified as Adler grade I (Figure 1C). Ultrasonic elastography indicated that the lesion exhibited intermediate stiffness (Figure 1D). Contrast-enhanced ultrasound revealed heterogeneous enhancement, with isoenhancement in partial central areas and absent or hypoenhancement across most regions (Figure 1E). The lesion was categorized as TI-RADS 3 and suspected to be a follicular neoplasm.

**Figure 1:**
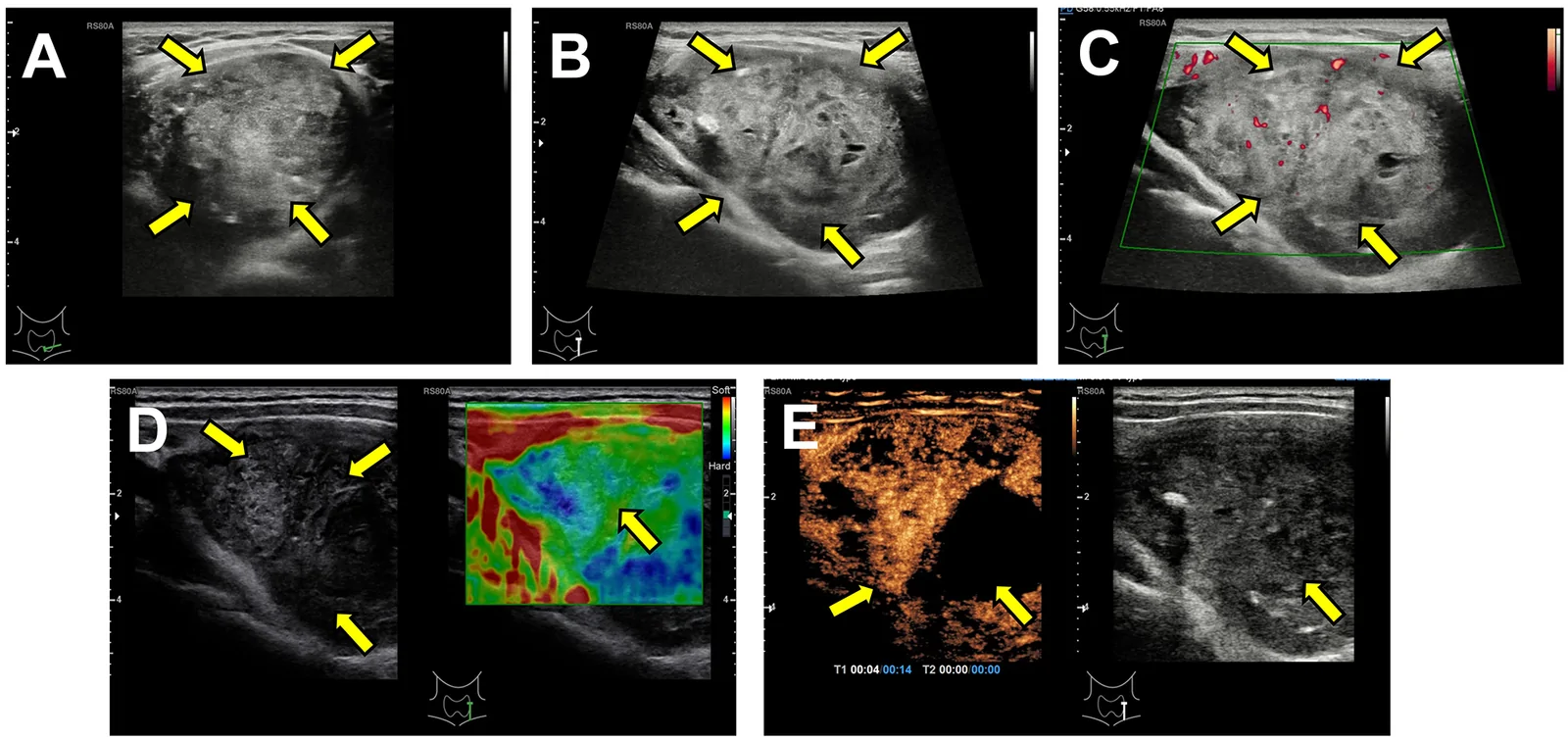
Ultrasonic manifestations. **(A,B)** B-mode ultrasound images showing a 62 × 35 × 47 mm mass with mixed echogenicity and relatively well-defined margins; **(A)** Transverse scan; **(B)** Longitudinal scan. **(C)** Color Doppler image demonstrating Adler grade I intralesional blood flow in the mass; **(D)** Ultrasound elastography showing the lesion with moderate stiffness; **(E)** Contrast-enhanced ultrasound image revealing heterogeneous enhancement.

CT displayed a slightly hypodense mass approximately 5.0 × 4.3 cm at the left root of the neck with scattered calcifications (Figure 2A). The lesion had ill-defined boundaries with the left thyroid lobe and marked heterogeneous enhancement with scattered intralesional vascular channels on contrast scanning, with tentative feeding supply from the left common carotid artery (Figure 2B). It caused compression and displacement of the adjacent trachea and esophagus, partially extended into the superior mediastinum, and compressed the brachiocephalic vein and proximal segments of left cervical vessels. Multiple cervical lymph nodes were visualized. The imaging findings were highly suggestive of a neoplastic lesion.

**Figure 2:**
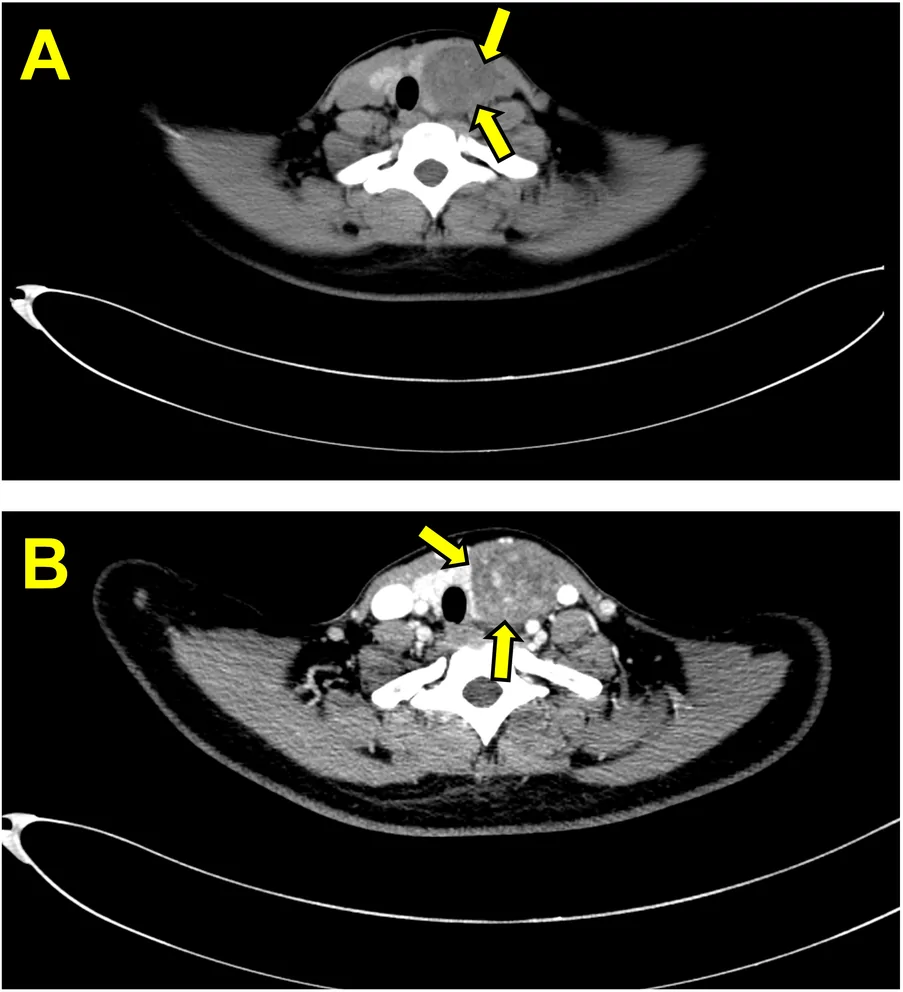
Neck CT findings. **(A)** Non-contrast scan; **(B)** Contrast-enhanced scan.

## Therapeutic intervention

Given the high preoperative suspicion of a thyroid neoplastic lesion, fine-needle aspiration (FNA) biopsy was performed, which failed to yield a definitive pathological diagnosis and yielded Bethesda category I. Left thyroid lobectomy combined with isthmectomy was performed. Intraoperative findings showed the tumor possessed an intact capsule. On cut section, the lesion was grayish-brown and predominantly solid with cystic components containing a small amount of old dark brown cystic fluid. The tumor was closely adherent to the capsule of the left thyroid lobe with mild adhesion to surrounding tissues.

## Outcomes and follow-up

Hematoxylin-eosin staining revealed densely packed fascicles of abundant spindle-shaped tumor cells, and no tumor infiltration was identified within the thyroid parenchyma (Figure 3A, B). Immunohistochemical (IHC) results were as follows: S100 (+), SOX10 (+), partial positivity for D2-40, CD117 (-), CD34 (-), DOG1 (-), Desmin (-), SMA (-), scattered Ki-67 (MIB-1) positivity in less than 1% of cells, Pan-CK (-), and retained H3 K27Me3 expression without loss. These pathological findings confirmed the diagnosis of schwannoma.

**Figure 3:**
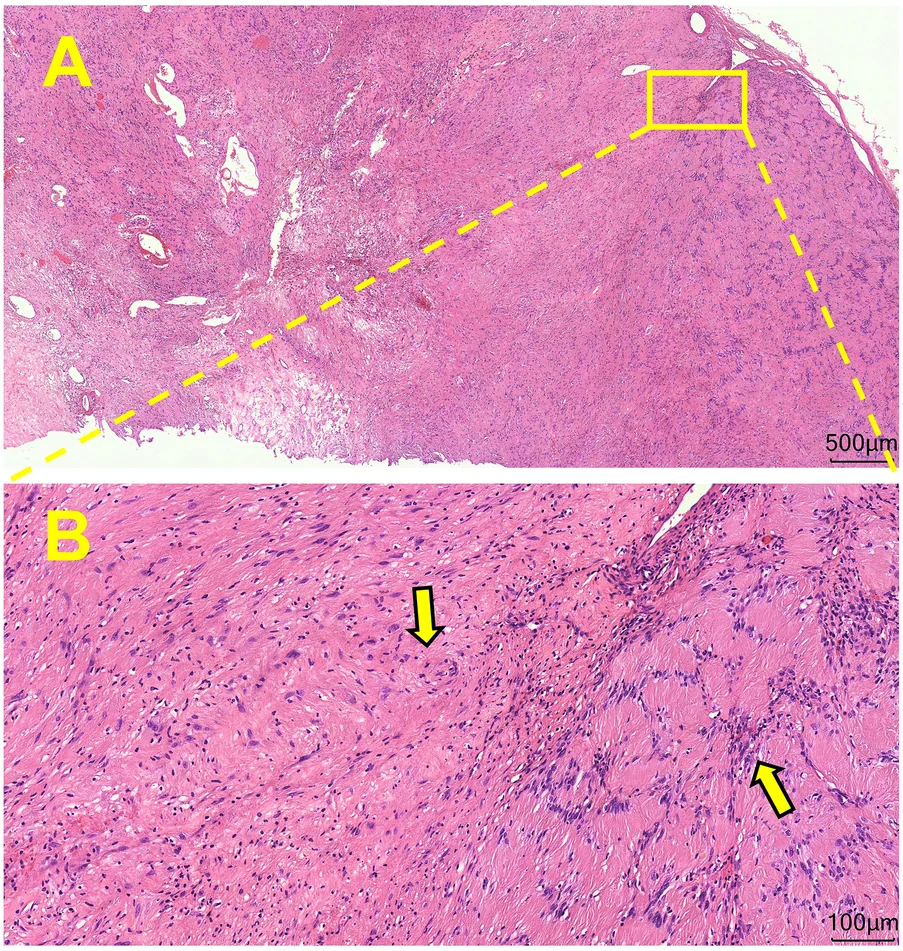
Histologic findings of the tumor. **(A)** Low-power overview of the lesion, with the yellow box denoting the magnified area shown in B (scale bar = 500 *μ*m). **(B)** Intermediate-magnification HE-stained image demonstrating fascicular spindle-cell region and marked collagenous degeneration (scale bar = 100 μm). Down-directed arrow: fascicles of spindle-shaped neoplastic Schwann cells. Up-directed arrow: hyalinized collagen-rich nodules.

The patient received serial ultrasound follow-up for one year postoperatively. No abnormalities were detected on laboratory tests or thyroid imaging examinations during follow-up.

## Discussion

Schwannomas occur across all age groups and follow an indolent clinical course. Primary thyroid region schwannomas are exceedingly rare (15–18), and those arising in the thyroid bed are even rarer. These lesions may originate from the sympathetic chain, glossopharyngeal nerve, vagus nerve, accessory nerve, recurrent laryngeal nerve, or hypoglossal nerve (3). In the present case, however, the exact nerve of origin could not be definitively identified intraoperatively. Ultrasonography is the first-line modality for evaluating thyroid nodules. Because thyroid-bed schwannomas grow adjacent to the thyroid gland, they are frequently misidentified as intrinsic thyroid neoplasms on ultrasound (3, 6, 7, 16, 19–24). This overlap is unsurprising and represents a major contributor to diagnostic confusion. The ultrasonographic features include well-circumscribed and regular morphology, predominantly hypoechoic parenchyma with homogeneous or heterogeneous internal echoes, cystic change, thickened walls, and rich intralesional and peripheral vascularity (3, 5, 25–28).

In the present case, preoperative ultrasonography revealed a 62 mm  ×  35 mm  ×  47 mm cystic-solid mass in the left thyroid region. The lesion showed transverse expansile growth, relatively well-defined margins, heterogeneous echogenicity with coarse calcification, and Adler blood flow grade 1. Contrast-enhanced ultrasound demonstrated heterogeneous enhancement, with isoenhancement centrally and absent or hypoenhancement in a large area, and no enlargement of bilateral cervical lymph nodes. A preoperative diagnosis of follicular neoplasm was tentatively made based on these morphologic features. Follicular epithelial-derived thyroid tumors, represented by follicular adenoma, demonstrate characteristic pathological and imaging features. These lesions are commonly associated with follicular dilatation, hemorrhagic cystic degeneration, and dystrophic calcification. On ultrasonography, they typically appear as well-circumscribed cystic-solid nodules with frequent coarse calcification. On contrast-enhanced ultrasound, solid components exhibit abundant vascularity whereas cystic degenerative areas show no enhancement. This imaging pattern is highly consistent with the findings in the present case (29, 30). While ultrasonography provides morphologic clues to differentiate follicular adenoma from follicular carcinoma, with the latter typically showing heterogeneous echogenicity, ill-defined or lobulated margins, and calcification (29–31), preoperative ultrasound only supports a morphologically based tentative diagnosis. It cannot definitively distinguish benign from malignant follicular neoplasms, nor can it accurately determine the anatomical origin of the lesion.

Lesion volume was calculated using the standard ellipsoid formula, defined as the product of anteroposterior, transverse and longitudinal diameters multiplied by π/6 (32, 33). In this case, the estimated lesion volume was approximately 78.8 cm³ at the 3-year follow-up before admission, and decreased to 53.4 cm³ at admission. The maximum longitudinal diameter increased from 58 mm to 62 mm over the 3-year interval, corresponding to a relative increase of 6.9%. Prior evidence shows that for well-circumscribed thyroid nodules larger than 1 cm, volume changes below 13.1% and maximum diameter changes below 7.3% on ultrasound should not be interpreted as true biological progression (32). Apparent volumetric discrepancies across serial examinations mainly stem from variations in scanning planes, measurement axis alignment, and intraobserver and interobserver variability. These are common limitations of serial ultrasound measurements for cystic-solid lesions and should be considered during follow-up (32, 33). Furthermore, cystic schwannomas may undergo spontaneous volume reduction due to absorption of intralesional cystic components and remodeling of degenerated tissue, which is a key cause of diameter and volume fluctuations during follow-up (34). A small subset of cases present with ill-defined borders with or without calcification (6, 23, 35). Calcification is rare in schwannomas, and when present, it often leads to misclassification as a malignant thyroid nodule (6).

These non-specific sonographic features make differential diagnosis from common thyroid nodules highly challenging. In this case, the tumor was located in the thyroid bed and closely abutted the left thyroid capsule. Preoperative ultrasound misclassified it as an intrathyroid nodule due to indistinct anatomical boundaries. Intraoperative examination also misjudged its origin as the left thyroid lobe due to capsular adhesion. Final pathology confirmed thyroid-bed schwannoma. This case fully illustrates the core diagnostic challenge that neither preoperative imaging nor intraoperative gross inspection can accurately determine the tissue origin of the lesion (3).

FNA is the first-line invasive diagnostic method for thyroid nodules. The overall preoperative diagnostic efficacy of FNA for thyroid region schwannomas is limited. Most cases fail to yield a definite histological diagnosis, frequently leading to misinterpretation as common thyroid nodules or nodular goiter, or merely indeterminate benign results with limited clinical utility (2, 5, 8, 9, 12, 27, 28). Indeed, it only achieves a diagnostic sensitivity of 0%–40% for schwannomas, with an inadequate specimen rate as high as 36%–50%, resulting in very limited diagnostic performance (27, 36). In contrast, ultrasound-guided CNB yields intact tissue cores that preserve histological architecture and enable immunohistochemical testing. It boasts high diagnostic accuracy for various head and neck neoplasms, and is especially suitable for differentiating mesenchymal tumors such as schwannomas (37, 38). International guidelines also recommend CNB as a routine diagnostic approach for cervical masses (39). Preoperative definitive diagnosis of thyroid-bed and lateral cervical schwannomas can be achieved by ultrasound-guided CNB (4, 40). For lesions located in the retroperitoneum, pelvis and other sites, transvaginal ultrasound-guided CNB can also confirm benign schwannoma before surgery (41). Some cervical masses are easily misdiagnosed as lymph node metastasis in patients with a prior history of malignancy, whereas CNB can establish a pathological diagnosis of schwannoma and prevent unnecessary anti-tumor therapy (42). Complete surgical resection remains the primary treatment for schwannoma, with a generally favorable prognosis reported in previous literature. Unlike intrathyroidal schwannomas, thyroid-bed schwannomas can be enucleated without thyroidectomy (6, 7, 20, 43). Hemithyroidectomy is required when the lesion is inseparable from thyroid tissue, and total thyroidectomy is only indicated in extremely rare cases (43, 44). Accurate preoperative diagnosis helps guide conservative surveillance or thyroid-sparing tumorectomy, thereby avoiding overtreatment (40).

## Conclusion

Therefore, for lesions with atypical imaging features, indeterminate fine-needle aspiration results or suspected extrathyroidal origin, multidisciplinary collaboration among cytopathology, radiology and surgical departments is recommended to establish a definitive preoperative diagnosis, and CNB can be performed to guide appropriate therapeutic strategies.

## Data Availability

The original contributions presented in the study are included in the article/Supplementary Material, further inquiries can be directed to the corresponding author.
